# Efficacy Evaluation of 3D Navigational Template for Salter Osteotomy of DDH in Children

**DOI:** 10.1155/2021/8832617

**Published:** 2021-05-22

**Authors:** Jie Yu, Qiang Shi

**Affiliations:** ^1^Department of Orthopedics, Henan Provincial People's Hospital, People's Hospital of Zhengzhou University, Clinical Medicine School of Henan University, Zhengzhou, China 450003; ^2^Department of Sports Medicine, Xiangya Hospital, Central South University, Changsha 410008, China; ^3^Key Laboratory of Organ Injury, Aging and Regenerative Medicine of Hunan Province, Changsha 410008, China

## Abstract

**Background:**

The aim of this study is to retrospectively evaluate the efficacy of 3D navigational template for Salter osteotomy of DDH in children.

**Methods:**

Thirty-two consecutive patients with DDH who underwent Salter osteotomy were evaluated between July 2014 and August 2017, and they were divided into the conventional group (*n* = 16) and navigation template group (*n* = 16) according to different surgical methods. The corrective acetabular degrees, radiation exposure, and operation time were compared between the two groups.

**Results:**

No nerve palsy or redislocation was reported in the navigation template group. Compared with the conventional group, the navigation template group had the advantages of more accurate acetabular degrees, less radiation exposure, and shorter operation time (*P* < 0.05). Meanwhile, the navigation template group achieved a better surgical outcome than the conventional group (McKay, *P* = 0.0293; Severin, *P* = 0.0949).

**Conclusions:**

The 3D navigational template for Salter osteotomy of DDH is simple and effective, which could be an alternative approach to improve the Salter osteotomy accuracy and optimize the efficacy.

## 1. Background

Developmental dysplasia of the hip (DDH), which is also called congenital hip dislocation, always results in osteoarthritis of the hip joint in adult life [1]. Salter osteotomy has been demonstrated to be one of the most effective surgical procedures for treating developmental dysplasia of the hip in children, which can not only increase the weight-bearing area of the acetabulum but also change the moment of forces acting on the hip joint by shifting the center of the femoral head [2]. However, performing Salter osteotomy accurately remains difficult because conventional imaging methods, such as X-ray or CT (computed tomography), were not sufficient to provide precise information about these complex deformities. Moreover, the success of osteotomy procedure always depends on personal clinical experience of the surgeon, which includes both excellent preoperative planning and intraoperative skills. Any error of preoperative planning or intraoperative manipulation will affect surgical outcomes of Salter osteotomy [3]. Therefore, a more simple and precise surgical procedure is needed.

Currently, navigational templates using 3D technology have been successfully applied in the field of orthopedic surgery [4–7]. To achieve ideal correction effects for Salter osteotomy, a new method that could facilitate the accurate osteotomy in DDH patients is needed. In this study, we utilized both 3D printing technology and reverse engineering for Salter osteotomy in children with DDH. Moreover, a novel patient-specific navigational template which allows to be firmly and accurately attached to the acetabulum was also designed and applied during the surgery.

Therefore, the aim of this study was to evaluate the efficacy of navigational template created by 3D printing technology for Salter osteotomy of DDH. Additionally, the prognosis and safety were also assessed compared with the conventional surgery retrospectively.

## 2. Materials and Methods

### 2.1. Patients

A total of 32 consecutive patients (22 female and 10 male) underwent Salter osteotomy were included in our department between July 2014 and August 2017. According to different surgical procedures, patients were divided into the conventional group (*n* = 16) and navigation template group (*n* = 16) by the random number table method. We excluded dysplasia of the hip joint or Tönnis grade I in this study, and there is no significant difference between the two groups in terms of age, gender, side, Tönnis classification, surgery history, and mean follow-up (Table 1). This study was approved by the Ethics Review Committee of Xiangya Hospital Central South University. Information was provided, and a consent form was signed by each patient before the study.

### 2.2. Osteotomy Angle Design and Navigation Template Fabrication

**Figure 1:**
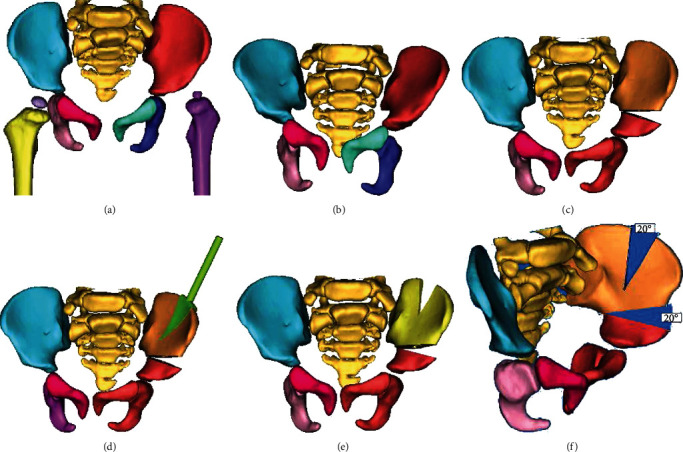
Design of navigational template for Salter osteotomy. (a–c) Simulate the angle of osteotomy using Imageware software. (d) The navigation template was designed with reverse modeling. (e, f) The corrective degree was simulated in the computer.

All patients underwent CT scan of the pelvis, and the image data were imported into the Mimics 20.0 software (Materialise, Leuven, Belgium) in DICOM (Digital Imaging and Communications in Medicine) format for 3D reconstruction. The fully interactive 3D reconstruction based on CT imaging data allowed full visualization of the pelvis. The osteotomy plane and accurate planning for the corrective degrees were defined based on the pelvic parameters. Then, the navigational template was designed on Geomagic Design X software based on the profiles of Salter osteotomy and manufactured by 3D printing technology (Figure 1). System parameters included the thickness of the processing layer at 0.1 mm and processing speed at 500 mm/s. The entire process of navigational template construction required approximately 6-15 h, with an average of 7.6 h. Ultimately, the navigational template was sterilized using low-temperature plasma before the surgery.

### 2.3. Surgical Procedure

A small sand bag was placed under the affected acetabulum when the patient was lying supine on the fluoroscopic operating table. All patients underwent open reduction and Salter osteotomy by one senior orthopedic surgeon in our department through an anterolateral approach (derotational femoral shortening osteotomy was performed if necessary). The navigation template was sterilized and applied intraoperatively to assist.

**Figure 2:**
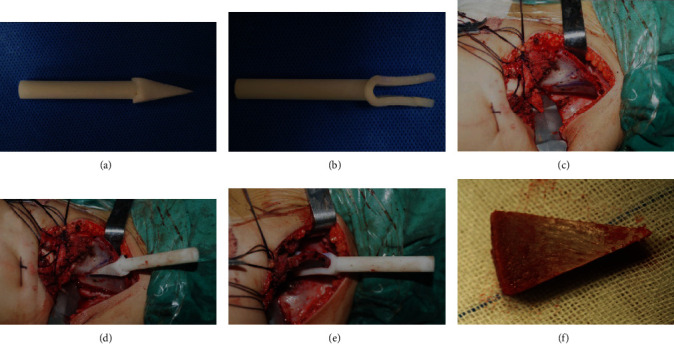
The navigation template and intraoperative operation. (a, b) The template was produced by 3D printing technology. (c–e) The template was installed on the ilium intraoperatively. (f) The wedge-shaped osteotomy block.

**Figure 3:**
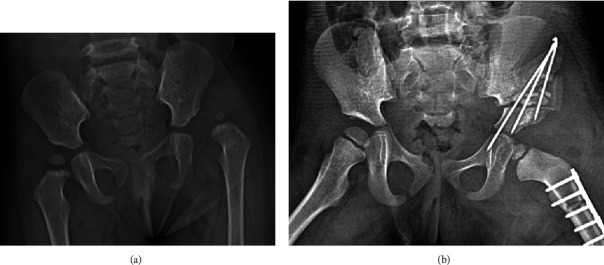
Preoperative and postoperative imaging data of a 25-month-old girl with DDH. (a) Anteroposterior radiograph of the pelvic before surgery. (b) Anteroposterior radiograph of the pelvic after surgery.

Salter osteotomy was performed on the basis of the preoperative simulation (Figure 2). After a triangular graft from ilium was taken using the novel navigational template, accurate Salter osteotomy was performed as preoperatively simulated and postoperative images revealed internal fixation with Kirschner wires (Figure 3). In the conventional group, the Salter osteotomy was performed by means of preoperative and intraoperative measurements.

### 2.4. Postoperative Management

Postoperative management procedures after Salter osteotomy were the same between the two groups. The spica cast fixation was used for 8 weeks and then changed to double lower limb brace with abduction and rotation of the hip for another 8 weeks. Moreover, radiographs of the pelvis were taken regularly to assess the degree of correction and signs of union until the region of Salter osteotomy completely healed, and then the Kirschner wires were removed. The mean follow-up was 3.1 years (2 to 5 years), and patients were reviewed at 8 weeks, 6 and 12 months, and then every year until skeletal maturity. Surgical outcome was assessed using the McKay clinical classification system [8] and the Severin radiographic scale [9] at a final follow-up.

### 2.5. Statistical Analysis

All measurement data in this study were statistically analyzed by the SPSS 25.0 software (SPSS, Inc., Chicago, USA) and manifested as count (percentage) or mean ± standard deviation (SD). Student's *t*-test, chi-squared test, and Fisher's exact test were applied to analyze the data in this study. Different parameters measured between the two groups were evaluated with an independent *t*-test for continuous variables and chi-square test or Fisher's exact test for the categorical variables. Statistical significance was set at *P* < 0.05.

## 3. Results

Regarding the mean follow-up period, there was no statistical significance between the conventional group (2.9 ± 0.8 years) and navigation template group (2.7 ± 0.5 years, *P* = 0.551).  Table 2 shows the clinical results of patients in two groups. As for the corrective AI (acetabular index) degrees, there was a statistical significance between the conventional group (24.6 ± 4.1°) and navigation template group (21.4 ± 2.2°, *P* = 0.0083). Meanwhile, the operation time in the navigation template group (25.6 ± 2.1 min) was significantly less than that in the conventional group (44.1 ± 3.2 min, *P* < 0.0001). According to the McKay standard, 14 patients in the navigation template group obtained excellent correction, 1 good, and 1 fair, while 6 patients obtained excellent correction, 4 good, 3 fair, and 3 poor in the conventional group. As a consequence, the navigation template group achieved a better surgical outcome than the conventional group (McKay, *P* = 0.0293; Severin, *P* = 0.0949; Table 2).

The leg-length inequality, hip range of motion, and gait pattern were measured for clinical assessment. Except that one patient was found to have slight avascular necrosis of femoral head, no nerve palsy or redislocation was noted in the navigation template group at the last follow-up.

## 4. Discussion

Open reduction and Salter osteotomy have been proved to be an effective method for patients with DDH between the ages of 2 and 6 years [10, 11]. In this procedure, the acetabulum is redirected and the distal segment is shifted downwards for correcting the anterolateral deficiency of DDH. The degree of acetabular index decreases as the gap is inserted by the triangular graft from the ilium. However, inadequate correction or overcorrection after Salter osteotomy may result in serious complication, such as subluxation, persistent instability, or posterior dislocation [12]. In this current study, we achieved ideal results in 16 cases of DDH with the assistance of novel navigational templates for Salter osteotomy.

At present, it is still a great challenge for the surgeons to accurately adjust the correction angle of triangular graft during Salter osteotomy because 2D or 3D CT images alone cannot provide sufficiently accurate information [3]. Furthermore, the procedure often needs repeated adjustment intraoperatively, which may result in the great deviation and poor outcomes. Newly developed medical equipment, such as 3D navigation or robot, is very promising and useful for the accuracy of surgery [13, 14]. Nevertheless, they are expensive equipment and time-consuming procedures and have complexity of the registration frame, which limits their use in most hospitals.

Until now, navigational templates have been widely used in various fields such as joint or trauma [15, 16]; however, only a few applications in pediatric orthopedic disorders have been reported [17]. Previously, various navigational templates had been made for cubitus varus and the clinical results were wonderful [18–20]. Lu et al. [21] validated the accuracy and safety of a novel patient-specific template in congenital scoliosis. With the assistance of 3D printing technology, it is the first time to provide a novel navigational template for Salter osteotomy to decrease the acetabular index for improving the reduction of femoral head and acetabulum. Moreover, unlike other navigational templates, the bar in the present navigational template was like a chip locked the ilium to avoid sliding errors. Hence, it was simple and easy for surgeons to perform Salter osteotomy without special training. According to the MacKay standard, 93.8% had excellent and good results in the navigation template group.

At last, it is still necessary to point out several limitations in this study. Firstly, the number of cases in our group is small and the present research is a simple retrospective study; more cases and multicenter prospective studies are needed in the future. Besides, the follow-up time of this study is relatively short that it is necessary to prolong the follow-up time because patients in the two groups were still not at the stage of skeletal maturity. Despite this limitation, the novel patient-specific navigational template in this study provides a new accurate way for Salter osteotomy. It is necessary to further improve the follow-up for a longer time.

## 5. Conclusion

The novel navigational template for Salter osteotomy using 3D printing technology fulfills the accuracy and reliable correction demands for Salter osteotomy of DDH in children, which might be an optional method to promote the accurate osteotomy and optimize the efficacy. Compared with the conventional group, the navigation template group has the advantages of higher rate of excellent correction, less radiation exposure, and shorter operation time.

## Figures and Tables

**Table 1:** Comparison of demographic data between two groups.

Characteristics	Conventional group (*n* = 16)	Navigation template group (*n* = 16)	*P* value
Mean age (range) (years)	3.7 ± 1.7 (2-6)	3.9 ± 1.8 (2-6)	0.678
Gender, *n* (%)			0.694
Male	5 (31.2)	4 (25.0)	
Female	11 (68.8)	12 (75.0)	
Side, *n* (%)			0.719
Left	9 (56.3)	10 (62.5)	
Right	7 (43.7)	6 (37.5)	
Tönnis classification			0.705
II	3 (18.8)	4 (25.0)	
III	8 (50.0)	9 (56.2)	
IV	5 (31.2)	3 (18.8)	
Surgery history			0.465
Yes	7 (43.7)	5 (31.2)	
No	9 (56.3)	11 (68.8)	
Mean follow-up (years)	2.9 ± 0.8 (2-5)	2.7 ± 0.5 (2-4)	0.551

**Table 2:** Comparison of operation data and functional outcomes between two groups.

	Conventional group (*n* = 16)	Navigation template group (*n* = 16)	*P* value
Corrective AI degrees (°)	24.6 ± 4.1	21.4 ± 2.2	0.0083
Radiation exposure (times)	6.9 ± 2.6	4.8 ± 1.9	<0.0001
Operation time (min)	44.1 ± 3.2	25.6 ± 2.1	<0.0001
Severin radiological results, *n* (%)			0.0949
Excellent	7 (43.8)	13 (81.25)	
Good	6 (37.5)	1 (6.25)	
Fair	2 (12.5)	2 (12.5)	
Poor	1 (6.2)	0	
McKay standard, *n* (%)			0.0293
Excellent	6 (37.5)	14 (87.5)	
Good	4 (25.0)	1 (6.25)	
Fair	3 (18.75)	1 (6.25)	
Poor	3 (18.75)	0	

## Data Availability

The data used to support the findings of this study are available from the corresponding author upon request.

## Abbreviations

3D: Three-dimensional
CT: Computed tomography
DDH: Developmental dysplasia of hip
DICOM: Digital imaging and communications in medicine
AI: Acetabular index.
